# Case Conceptualizing in Acceptance and Commitment Therapy for Moral Injury: An Active and Ongoing Approach to Understanding and Intervening on Moral Injury

**DOI:** 10.3389/fpsyt.2022.910414

**Published:** 2022-06-30

**Authors:** Lauren M. Borges, Sean M. Barnes, Jacob K. Farnsworth, Kent D. Drescher, Robyn D. Walser

**Affiliations:** ^1^Rocky Mountain Mental Illness Research, Education and Clinical Center for Veteran Suicide Prevention, Aurora, CO, United States; ^2^Department of Psychiatry, University of Colorado Anschutz Medical Campus, Aurora, CO, United States; ^3^Rocky Mountain Regional VA Medical Center, Aurora, CO, United States; ^4^National Center for PTSD, Palo Alto, CA, United States; ^5^Department of Psychology, University of California, Berkeley, Berkeley, CA, United States

**Keywords:** moral injury, Acceptance and Commitment Therapy (ACT), veterans, health care providers, functional contextualism

## Abstract

Acceptance and Commitment Therapy for Moral Injury (ACT-MI; 10–11), is an application of Acceptance and Commitment Therapy principles designed to help individuals live their values, even in the presence of moral pain. ACT-MI differs from other emerging treatments for moral injury in that ACT-MI is not based on a traditional syndromal approach to conceptualizing moral injury, which treats moral injury as a collection of signs and symptoms to be reduced. Rather than assuming moral injury causes suffering through a constellation of symptoms that a person *has*, in ACT-MI, moral injury is defined by what a person *does* in response to moral pain. Consistent with this framework, we present a unique approach to moral injury case conceptualization that emphasizes function over form, providing clients the opportunity to break free from the patterns of behavior that cause moral injury-related suffering to persist. Rooted in approaches to conceptualizing that have demonstrated utility in extant interventions (e.g., ACT), ACT-MI clinicians conduct ongoing functional analyses to inform case conceptualization and intervention. Functional analysis is used to disrupt the processes maintaining moral injury, as the client and therapist work to identify and intervene on the behaviors reinforcing avoidance and control of painful internal experiences causing moral injury. In the current article, we guide the reader through a framework for applying functional analysis to the conceptualization of moral injury where the reinforcers driving moral injury are explored. We also provide examples of questions that can be used to help uncover the functions of moral injury consistent behavior. Case examples based on our experiences treating moral injury are presented to demonstrate how various types of morally injurious events can evoke different features of moral pain which in turn motivate different repertoires of avoidance and control. These inflexible patterns of avoidance and control create suffering by engaging in behavior designed to escape moral pain, such as social isolation, spiritual disconnection, reduced self-care, suicidal ideation, and substance use. We discuss how to target this suffering using functional analysis to guide treatment decisions, matching interventional processes within ACT-MI to the specific functions that moral injury-related behavior is serving for an individual. We suggest that the use of functional analytic case formulation procedures described herein can assist clients in disrupting behavioral patterns maintaining moral injury and thereby free them to pursue lives of greater meaning and purpose.

## Introduction

### Conceptualizing Moral Injury From a Syndromal Perspective

Moral injury is at a critical juncture in its conceptualization. There is no agreed upon definition for moral injury, no diagnosis for moral injury in the Diagnostic and Statistical Manual for Mental Disorders (DSM), no consensus regarding measures or cut points to determine the presence or absence of “moral injury,” and disagreement about the components of moral injury that are critical for assessment and intervention. Researchers have noted several varying definitions of moral injury and the need for uniformity regarding a definition, however, there is still no authoritative definition of the construct (1–5). While moral injury is not yet characterized as a diagnostic syndrome in the DSM, the mental health community is moving toward a syndromal model of conceptualizing moral injury (1, 6). Syndromal models of moral injury include conceptualization that classifies experiences like guilt and shame as symptoms causal to psychopathology (3–5), a focus on creating measures to identify signs and symptoms, establishing cut points for these measures indicating the presence or absence of moral injury, and targeting the reduction of these symptoms in treatment (7–10). Syndromal approaches to conceptualization focus on understanding the topography of a psychological disorder and its boundary conditions. In determining boundary conditions or cut points for moral injury, researchers hope to better understand when instances of moral injury are clinically significant and when there is not sufficient symptom severity to apply the clinical label.

Treatment following a syndromal conceptualization would entail an emphasis on reducing moral injury symptoms such that successful treatment results in “moral injury” no longer being diagnosed. Relying on the presence or absence of moral injury and focusing on these symptoms in treatment assumes that the experiences of the group for whom a scale was developed leads providers to the most salient variables to target for an individual. However, because clinical cut points are rooted in the statistical averages of large groups, such an approach may not be sensitive to the nuanced and contextual processes maintaining moral injury for specific individuals and therefore could serve as a barrier to efficient and effective psychotherapy (11, 12). Caution regarding the establishment of cut points for moral injury is warranted given that applying such methods have not historically clarified the etiology of other psychological syndromes, predicted the course of psychopathology, or facilitated treatment responsivity (11, 13, 14).

### Conceptualizing Moral Injury From a Functional Contextual Perspective

Functional contextualism is a philosophy of science rooted in pragmatism, where behavior is viewed as an “act in context.” Any analysis of a behavior is interpreted as an ongoing act inseparable from its current and historical context (15). As an alternative to a *syndromal* approach to conceptualization focused on assessing the symptom topography of moral injury, a *functional* approach to conceptualization is focused on understanding behaviors maintaining moral injury through the purpose(s) they serve for the individual (11, 13, 14, 16–19). Rather than focusing on reducing what would be conceptualized as symptoms of a moral injury disorder from a syndromal perspective (e.g., reducing self-blaming thoughts), from a functional contextual perspective it is the person’s relationship with their experiences (e.g., how they relate to self-blaming thoughts) that is emphasized in treatment. In this approach, practitioners are more concerned with the *functions* of a person’s behavior (i.e., What are the consequences that maintain the behavior?) and the *contextual factors* that give rise to these behaviors (i.e., Under what conditions does this behavior occur?) to understand “what is the purpose of this behavior for this individual in this context?”, rather than focusing on shifting the *form* or content of a person’s experience (e.g., Is the thought itself changing?).

#### Functional Contextual Definition of Moral Injury

Moral injury is a *pattern of behavior* defined by an individual engaging in efforts to avoid or control their moral pain. This avoidance and control behavior often functions to reduce and/or change a person’s experience of moral pain. Control efforts, for instance drinking alcohol, may temporarily decrease an individual’s experience of moral pain. However, these control efforts often lead to long-term consequences causing social, psychological, and spiritual suffering (17). From a functional contextual perspective, an individual’s specific pattern of moral injury is assessed through a functional analysis.

To assess moral injury, first it is necessary to identify the behavior that is causing the cycle of suffering in relating to their moral pain to persist. To do this, an individual’s experience of a potentially morally injurious event (PMIE), the event that violated their moral code, should be explored. PMIEs can occur through a person’s own actions, inactions, or through other peoples’ actions or inactions. These moral code violations may be particularly salient when they are experienced as being at odds with a person’s socially held values. For instance, PMIEs that violate a person’s religious or spiritual beliefs, sense of social justice, and ethics about what is right and wrong may be more likely to evoke distressing experiences. Determining a person’s PMIE exposure is an important step in understanding their experience of moral pain.

Moral pain can be defined as the thoughts (e.g., self-directed such as, “I don’t deserve to be happy” and other-directed such as, “I’ll never be able to forgive them”), emotions (e.g., guilt, shame, contempt, anger, and disgust), sensations (e.g., feeling of nausea), and urges (e.g., urges to isolate and hide from others) that exposure to the PMIE has evoked. Moral pain is assessed so that the client and therapist can understand how the individual responds to these internal experiences. Because moral emotions are painful and often aversive, responses to these experiences tend to include strategies that facilitate avoiding, fixing, or attempting to control one’s moral pain in some form. Attempts to avoid and control moral pain make sense, given social and verbal learning histories that support avoidance of painful internal content (e.g., “Don’t feel bad,” “Just get over it,” and “Be happy”). However, if these behaviors are the only strategies used in response to moral pain, they can profoundly impact a person’s life over time.

Suppose an individual experiences intense moral pain due to exposure to a PMIE, and consistently seeks to avoid these painful experiences, thus providing temporary relief. In that case, escape will be continuously reinforced, variation in behavior reduced, and opportunity for other sources of reinforcement eliminated (e.g., isolation in multiple contexts and for long periods reducing the possibility of experiencing other emotions such as joy). This could lead to additional difficulties connecting to sources of meaning and purpose. Social relationships, sacred practices, and even the individual’s relationship with themselves begins to suffer. Adding to this, suffering can expand and deepen as the strategies some individuals may use to avoid moral pain (e.g., attempting suicide) often create new sources of moral pain for both the person and their loved ones, thereby expanding and deepening the self-reinforcing cycle of avoidance and suffering.

From a functional contextual perspective taking into account the context in which a behavior is elicited and the purposes that behavior serves for the individual, behavior causing moral injury to persist occurs when an individual’s *relationship* to moral pain creates suffering. Furthermore, in conceptualizing painful moral emotions as serving important social and evolutionary functions (20–25), the presence of moral pain is not a signal of psychopathology, but a signal that an individual has experienced a moral code violation. Thus, rather than seeking only to reduce the pain, a functional approach seeks to address the underlying issues communicated by the pain. Therefore, instead of applying the label of “moral injury” to clients once their moral pain has crossed a certain subjective threshold, within a functional contextual framework “moral injury” is defined as the behavior(s) someone *does* in response to their moral pain that negatively impacts their ability to live a meaningful life. As noted, these behaviors tend to include avoidance, control, or doing something to temporarily fix an individual’s experience of that pain which can cause social, psychological, and spiritual suffering. An approach to treatment following this conceptual framework emphasizes changing the client’s relationship with their moral pain for the sake of building a life lived based on the client’s chosen values rather than a life centered around their reflexes to their moral pain.

**FIGURE 1:**
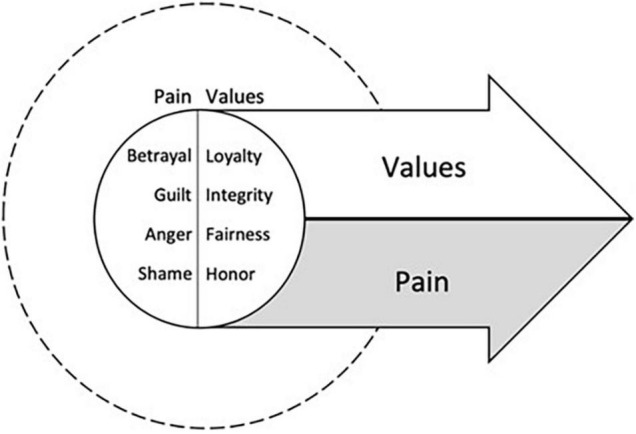
The treatment targets of Acceptance and Commitment Therapy for Moral Injury (ACT-MI), pursuing values in the presence of moral pain.

**FIGURE 2:**
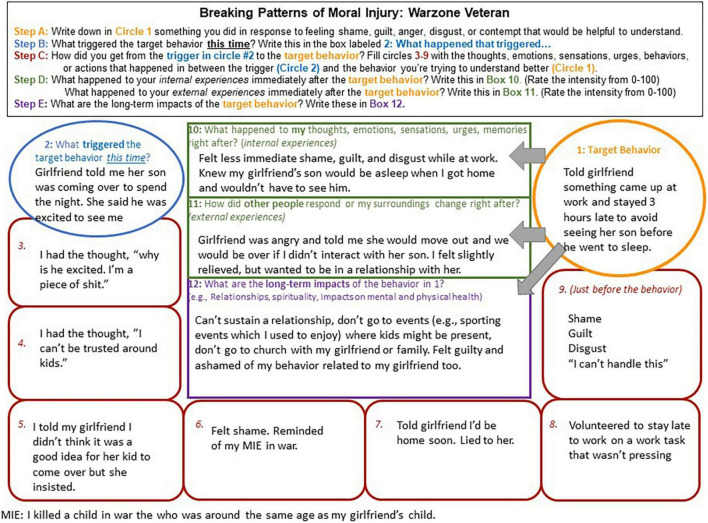
A warzone Veteran case example of a chain analysis demonstrating the factors maintaining moral injury.

**FIGURE 3:**
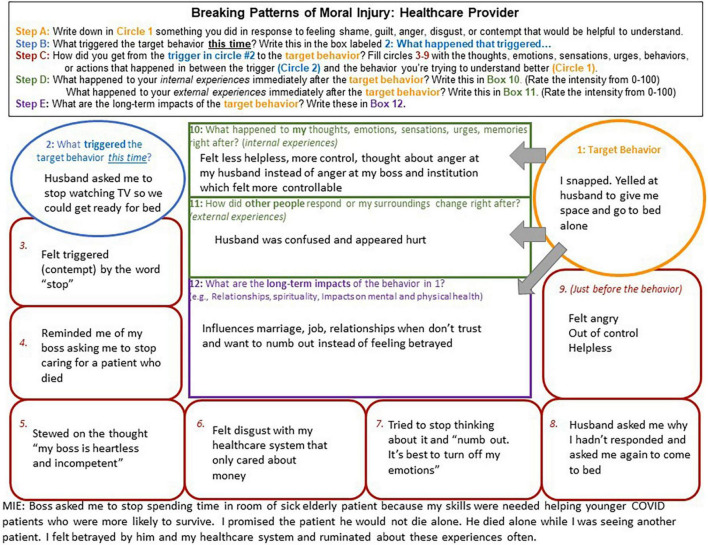
A healthcare provider example of a chain analysis demonstrating the factors maintaining moral injury.

In this article, we describe case conceptualizing in Acceptance and Commitment Therapy for Moral Injury (ACT-MI) (Figure 1), an approach to intervening on moral injury that is rooted in functional contextualism (17, 18). Rather than assuming moral injury causes suffering through a constellation of symptoms that a person *has*, in ACT-MI, moral injury is defined by what a person *does* in response to moral pain that interferes with living their values. We describe how functional analysis is implemented in ACT-MI and walk the reader through in-depth functional analysis via chain analysis and abbreviated, in-session functional analysis that occurs in the context of our therapy groups. Two detailed case examples are described, the first related to moral injury stemming from how a warzone Veteran related to his moral pain following a perpetration-based MIE (killing a child in war who was around the same age as his girlfriend’s child) involving a violation of his moral code (Figure 2). The second case example includes a health care provider who experiences a betrayal-based MIE from her supervisor and a betrayal by her institution prompting difficulties relating to her moral pain (i.e., a health care provider’s patient died alone of COVID-19 after she promised she would be there with him due to her boss and institution asking her to be elsewhere) (Figure 3). These cases provide examples of how functional analysis is used to guide assessment and treatment in ACT-MI.

### What Is Functional Analysis?

Functional assessment or *functional analysis* is central to behavioral therapy (19, 26, 27). To determine the functions of behavior which maintains moral injury, an assessment of the functions of that behavior is necessary. Extending the principles of behaviorism to understanding internal experiences including language and cognition in context, different interventions have been developed and organized targeting the relationships between an individual’s environment, covert, and overt experiences [e.g., Acceptance and Commitment Therapy (ACT), Dialectical Behavior Therapy (DBT), Process Based Cognitive Behavioral Therapy (PBCBT)]. Within these interventions, different methods have been implemented for understanding complex networks of thoughts, emotions, sensations, memories, urges, and behaviors to facilitate functional analysis (28–30). All methods of functional analysis include identification of a behavior maintaining suffering, the function(s) that behavior serves, and the contexts in which the behavior occurs. Each of these components is critical to not only understanding *why* an individual is suffering, but to facilitating intervention on the processes *maintaining* moral injury.

### How Functional Analysis Is Applied in Acceptance and Commitment Therapy for Moral Injury

Functional analysis is a core component of assessment and treatment in ACT-MI. In addition to previous applications of ACT-MI in individual contexts (18), ACT-MI is also currently under investigation for individuals engaged in group psychotherapy. To participate in moral injury treatment in a psychotherapy group more effectively, throughout treatment the individual client and therapist must clearly understand the behaviors contributing to the client’s moral injury. Since moral emotions are social experiences, we hold that learning new skills to relate to moral pain in the presence of other Veterans provides more opportunity to practice psychological flexibility and build a social community (17, 20). Group members can share their avoidance experiences and the costs and the benefits of changing their relationship to moral pain. Compassion and empathy for the costs of control can be validated by others while also supporting values-based behavioral change.

The group ACT-MI intervention also includes both individual functional analysis and in-session functional analysis in the therapy groups. There are three individual sessions for each group member devoted to case conceptualizing in ACT-MI: (1) the first before beginning the treatment group, (2) the second midway through treatment, and (3) the third after the final group session to promote skills generalization following treatment. These three sessions are devoted to functional analysis of behaviors maintaining moral injury.

Functional analysis also continues in group therapy sessions. These functional analyses are often abbreviated to fit into the allotted time, allowing patterns maintaining moral injury to be targeted in the moment so that group members can practice flexibly interacting with moral pain together. To facilitate a shortened within-group session functional analysis, a behavior occurring during that session is identified which is hypothesized to be maintaining moral injury and the contextual factors occurring immediately before the behavior and consequences immediately following the behavior are explored. For example, if a discussion in group evokes shame and a member tells a joke in response to that shame, the group’s (and Veteran’s) attention might shift from the content evoking shame to the content of the joke, reducing contact with shame (escaping the emotion). In this way telling the joke is negatively reinforcing because it reduces the group’s experience of shame and the member(s) feel relief. However, this joking behavior does not facilitate the development of new skills to relate differently to that shame. Addressing the joking behavior in session helps determine the function of behaviors that maintain moral injury and teaches group members how to do a functional analysis of their own behavior, examining the impact and workability of the consequences. Additionally, engaging functional analysis within a group context can be helpful in highlighting shared experiences across group members (e.g., similar avoidance or control behaviors, similar experiences of moral pain, similar costs of avoiding and controlling moral pain), facilitating group connection. Functional analysis is an ongoing process, engaged through multiple levels (individually and in group) that is designed to disrupt the patterns of behavior that maintain moral injury.

### Chain Analysis as a Method of Organizing Functional Analysis

In ACT-MI individual case conceptualizing sessions, chain analysis (a method of functional analysis most often associated with DBT) is used to organize the complex networks of behaviors, thoughts, emotions, sensations, memories, urges, and other events that can give rise to moral injury. Chain analysis provides the structure to identify the basic contingencies causing a behavior to persist (29, 31–33). In the following sections we describe the use of chain analysis to disrupt the patterns of behavior maintaining moral injury, and present two case examples to illustrate the application of chain analysis.

#### Starting With the Target Behavior

Because we contend that moral injury is not a syndrome existing *inside* of a person, but a pattern of behavior that someone *does* in response to moral pain which causes them suffering, individual actions [albeit external or internal (e.g., rumination)] become crucial sources of assessment data. Collaboratively choosing a behavior that the client and therapist believe could be maintaining moral injury is then a necessary starting point for a chain analysis. Without first identifying a specific behavior to assess, it is impossible to understand what reinforcing functions that behavior might serve and the contexts which might make the target behavior more likely. Specifying a target behavior that potentially maintains moral injury is therefore the first step toward the eventual goal of disrupting the cycle of moral injury-related suffering.

Several diagnostic syndromes and behaviors have been associated with exposure to potentially morally injurious events including PTSD, depression, substance use disorder, and suicidal ideation and suicidal behaviors (34–39). These syndromes and behaviors provide some clues about the kinds of behaviors that individuals may use to cope with their moral pain. For instance, research outside of the field of moral injury has demonstrated that experiential avoidance (avoiding experiences like moral pain) is connected to a number of the behaviors that are characteristic of moral injury. Experiential avoidance has been associated with suicidal ideation and behavior, substance use, and social isolation (40–42). These overt behaviors can be easily observed by others, often making them clear areas of focus as potential target behaviors that are maintaining moral injury.

In addition to behaviors that seem more obviously connected to efforts to manage moral pain, subtle behaviors are also important to assess. Behaviors that appear in their *form* or content to be productive or even consistent with a client’s values can nonetheless *function* as avoidance or control. While *any behavior* could serve the function of maintaining moral injury, supporting the need for idiographic assessment, overcorrecting and over-repairing are notably associated with moral injury. If someone engages in a behavior meant to repair a moral transgression, when they are participating in this reparative behavior, it could function as a means of avoiding or controlling their experience of moral pain. For instance, a client may donate to orphanages because children were harmed by their actions during their service in war. In the moments they are donating to the orphanage and shortly thereafter, the client might have less contact with their experience of guilt or shame, because they are focused on making amends and perhaps feel uplifted by donating. In the long term, however, if the donating behavior becomes a rule (e.g., “I must donate to repair”) and the individual is seeking relief repeatedly in the service of feeling better at the expense of other areas of meaning, then the donating behavior can be conceptualized as maintaining moral injury. The client may feel compelled to repeat donating each time they experience moral pain. Rather than serving as an actual values-based choice regarding amends, donating behavior serves an escape function for this client.

Furthermore, donating to an orphanage does not support the client in learning new skills to relate differently to their moral pain. Instead, they may encounter financial hardships or other hardships (e.g., loss of time with family) due to the desire to feel less shame. While the form or content of this behavior may appear productive and values-consistent and be socially rewarded, it is functioning to maintain suffering for this individual client. Regardless of the behavior’s form, if a client is engaging in that behavior for the purpose of “feeling better” it is possible that the behavior is maintaining moral injury.

Covert behaviors [e.g., the way a client interacts with their moral pain through behaviors not observable to others (for instance rumination, spending time trying to block a thought out)] may also cause moral injury to persist. For instance, a client who remembers killing a child in war might ruminate about what their involvement in this event means about themselves as a person. They may experience cycles of intentionally thinking thoughts like “I deserve to suffer,” justifying their current dilemma but also perpetuating unworkable behavior. A functional analysis explored by the client and therapist would help the client notice that when they ruminate on the thought “I deserve to suffer,” they also avoid having to directly contact the emotions associated with the memory of killing the child because instead they are focused on that thought. Rumination may serve to help them avoid the intensity of the pain of the incident, the pain of potential rejection by others, and suffice as self-punishment for past behavior. As another example, a client may have the same experience (killing a child in war). Instead of ruminating about a thought, they may mentally replay the details of the MIE over and over, attempting to problem solve the situation in a way that would lead to a different outcome (relinquishing the present moment to the past). Yet another possibility related to the same experience of killing a child in war could involve attempts to “block out” and “deliberately think about other things” when the thought “I deserve to suffer” appears. Blocking out and thinking about other things often causes rebound of the suppressed thought (43). This rebound can lead to even greater attempts to block the thought, and a vicious cycle ensues. Each of these scenarios would involve slightly different interventions to disrupt the patterns maintaining moral injury.

Over time, clients often develop complex behavioral networks including different strategies they use to avoid or control their moral pain. Several behaviors could be targeted through functional analysis and then intervened on using ACT-MI strategies. Determining which behaviors to target for intervention should always be done through collaborative discussion with the client. This said, initial target behaviors selected for intervention should ideally be those hypothesized as most impactful to the client’s life and that are directly connected to the client’s efforts to avoid or control moral pain. Although changing these behaviors will be more likely to elicit moral pain, it is helpful to explain to the client that starting with the highest impact behavior is also more likely to efficiently and effectively disrupt the networks maintaining moral injury, allowing the possibility for new learning to occur in the presence of moral pain.

To better understand how we apply chain analysis in ACT-MI, we first describe the components of chain analysis and then provide two case examples using functional analysis to facilitate assessment and intervention. In the first example (Figure 2), we describe a pattern maintaining moral injury for a Veteran whose MIE was related to killing a child in war.

We targeted this Veteran’s behavior of staying late at work to avoid interacting with his girlfriend’s son who reminded him of the child he killed in war. In the second example (Figure 3), we describe a pattern maintaining moral injury for a healthcare worker who is experiencing betrayal related to her institution whose target behavior was yelling at her husband (MIE of following her bosses’ orders which resulted in an elderly patient dying alone).

#### Triggering Events

After identifying a target behavior, it can be useful to identify factors that triggered the series of thoughts, emotions, sensation, urges, memories, and other behaviors that led to the target behavior. Given that moral pain is rooted in social experience, additional considerations are warranted. For instance, shame and guilt are emotions that are generally prompted by how an individual’s behavior has negatively impacted other people. In contrast, anger and contempt tend to arise from experiences of how other peoples’ behavior has impacted the individual and their communities. Therefore, events that trigger moral pain and subsequent attempts to avoid and control that pain are often interpersonal in nature. Particularly evocative triggering events might also be directly or indirectly tied to an individual’s experience of their MIE.

Examples of specific events that go on to trigger a pattern of moral injury-maintaining behaviors might include interpersonal disputes (e.g., a Veteran who was betrayed by an officer later has a conflict with their supervisor at work), other social interactions (e.g., a First Responder who felt helpless to prevent death being invited to attend a funeral for a family member), and direct reminders of a MIE (e.g., returning to work as a health care provider perceiving that they killed a COVID-19 patient). If the individual is unaware of what triggered their target behavior, the provider and client can work backward from the target behavior through the links in the chain until it is clear what led to the target behavior. Please see the case examples for examples of a triggering event for a warzone Veteran (Figure 2) and a healthcare provider (Figure 3).

#### Links in the Chain

Links in the chain include any facets of moral pain, behavior connected to that moral pain, and relevant contextual factors important to a person having engaged in the target behavior. This includes the client’s behaviors, events and interactions with others that are relevant contextual factors to the chain and may have prompted the client’s behavior, thoughts (e.g., self-blaming thoughts, thoughts related to blaming others), emotions (e.g., anger, shame, guilt, contempt, and disgust), sensations (e.g., pit in the stomach, tightness in the chest, and difficulty breathing) urges (e.g., urges to isolate and urges to attack), and memories (e.g., the memory of the MIE). If the client’s target behavior is indeed relevant to moral injury, then these links should always include such facets of moral pain. Furthermore, the links in the chain should form a complete narrative about the client’s target behavior that is maintaining their moral injury, spanning the triggering event to the target behavior. If the chain does not make sense, important links are likely missing. Collaboratively sharing your experience with the client (e.g., “Help me understand how you got from seeing a picture of your grandfather to attempting suicide”) may help uncover any factors that are important in maintaining moral injury. For detailed examples of links in the chain please see Figures 2, 3 for a Veteran and health care provider example.

#### Consequences

The immediate consequences of engaging in the target behavior are critical to uncover both in terms of how the behavior influences a person’s private experiences and how the behavior influences others in that person’s life. If the consequences maintaining moral injury can be directly addressed through behavior change, the pattern creating moral injury can be disrupted, and suffering reduced. In examining consequences, we are interested in understanding how the client’s thoughts, emotions, sensations, urges, and memories are impacted by the target behavior. In general, target behaviors tend to be maintained by experiential avoidance. This could take the form of negative reinforcement, wherein the target behavior facilitates escaping an aversive experience (e.g., thinking about suicide provides a sense of relief from current pain). Alternatively, positive reinforcement, where the target behavior facilitates contact with a preferred or more desirable experience, can also maintain moral injury (e.g., substance use, viewing pornography, and eating). Additionally, some target behaviors can be maintained through multiple processes (e.g., positive and negative reinforcement). For instance, isolating through video games could result in allowing someone to distract from shame and facilitate putting them in contact with emotional experiences that are more reinforcing than shame (e.g., anger, achievement, and excitement).

Responses from others can also influence moral injury. Continuing with the example of video games, consider a Veteran who finds spending time with their children aversive due to a previous MIE involving children. When the Veteran begins playing video games, their spouse keeps their children away from them in a well-intentioned attempt to help them control their moral pain through avoidance. This would be an example of negative reinforcement by the client’s environment. As clients experiencing cycles of moral injury are often prone to making explanations for their behavior based on their sense of self (e.g., “I must be an evil person”), identifying these sources of reinforcement is also important. This assists in understanding how to disrupt moral injury. It can also be validating for the client to understand reasons for their target behavior and in turn generate motivation for change.

In addition to understanding the immediate consequences of behavior maintaining moral injury, it is also important to understand the costs of engaging in these behavior patterns over time. Determining the long-term costs of moral injury patterns, and how these costs affect functioning and a person’s ability to engage their values (opportunities to connect with meaning and purpose) can also help facilitate motivation for behavioral change. We often see difficulties in functioning and disengagement from values manifest in interpersonal relationships, spirituality, and self-care as behaviors in these domains often result in opportunity for exposure to moral pain. For a person to see how their behavior departs from their values and how this behavior (e.g., isolating in relationships) has not decreased or eliminated their moral pain – and in some ways has created more pain (e.g., caused important relationships to end) – is important in creating motivation to interact differently with moral pain. Please see Figures 2, 3 for the short and long-term consequences of behavioral patterns maintaining moral injury.

### Abbreviated Functional Analysis

While formal chain analysis provides a comprehensive narrative about the behaviors maintaining moral injury, this level of detail may not always be feasible (e.g., in the context of a therapy group). However, the key components of functional analysis can still be applied to behaviors occurring within a group treatment session that are hypothesized to be maintaining moral injury. The key components of an abbreviated functional analysis involve assessing a behavior that facilitated experiential avoidance, the contextual factors in which the behavior occurred, and the consequences of that behavior. Essentially this approach is focused on understanding an antecedent, a behavior, and its consequences. While an abbreviated functional analysis may result in less depth, depending on the context it may facilitate greater opportunity to intervene on moral injury in the moment. Having completed more detailed chain analysis prior to group sessions may help to inform which of a specific group members’ behaviors are most relevant for facilitators to focus on for skills training.

### Questions to Elicit the Function of a Behavior

Whether engaging in a complete behavioral chain or abbreviated functional analysis, some specific questions can help identify the target behavior and uncover the function(s) of that behavior. In session, several behaviors can be observed that indicate processes related to experiential avoidance. When a noticeable shift occurs in the client’s affect, tone, behavior, or attention, it can be helpful to ask questions to understand their current experience and what happened immediately before the behavior of focus. For instance, if the client shifts from crying to expressing anger toward group members or the facilitator, asking the client a question like “*what happened just before you became irritated?, Did anything change about your sadness when you became mad?”* may help to uncover what the purpose of their “angry” behavior was. These kinds of questions can help to assess what happened immediately before and after the target behavior maintaining moral injury.

If someone’s behavior involves a less noticeable emotional shift, other kinds of questions may be helpful, such as questions that facilitate assessing the client’s attention. Questions like *“where did your mind go just then?”* could reorient the client back to the room and allow for more information to be gathered about the functions of their attentional shift. Even responding to changes in body language can be useful in assessing and intervening on the behaviors maintaining moral injury. For example, suppose a client suddenly seems closed off to the group (e.g., hunched over, averting eye contact) in their body posture, a provider could comment on this behavior, stating “*I’m noticing you are sitting like this*…*what are you experiencing right now?”*. If there are changes noticed within an ACT-MI psychotherapy group, asking group members for their experiences of the behavior or other trigger that created a shift for the group can also be useful.

### Intervening With Acceptance and Commitment Therapy Processes Using the Functional Analysis

One of the most critical facets of functional analysis is its direct treatment utility. This occurs when functional analysis helps the clinician identify ACT processes in the moment, applying interventions immediately and for use in future situations assisting the client to break free from patterns of moral injury occurring in and out of session. ACT-MI is based on the principles of ACT, but explicitly focused on targeting flexible responding to moral pain. The primary purpose of ACT-MI is to use processes related to acceptance (contacting the present moment, defusion, acceptance, self as context) and change (values, committed action) to empower clients to move toward their values even in the presence of moral pain (Figure 1) (17, 18). While the purpose of this article is not to comprehensively describe ACT-MI, more detail is provided in the section that follows about how the processes of contacting the present moment, defusion, acceptance, self as context, values and committed action can be facilitated using functional analysis to disrupt the patterns of suffering maintaining moral injury.

Within ACT-MI sessions, engaging in functional analysis typically involves the client directly contacting moral pain and interacting with that pain flexibly (e.g., noticing moral pain, describing it to a provider/group). In this way participating in a functional analysis in a therapy session can evoke several treatment processes within ACT-MI [e.g., contacting the present moment through noticing one’s experiences in the here and now, observing that experience and describing it through defusion, making space for moral pain to exist as an experience that you have rather than an experience you are through acceptance, stepping back from narratives about the need to control moral pain through self as context, interacting differently with moral pain to engage values (e.g., genuine in relationships, learning about myself), and practicing committed action related to values (e.g., engaging in an exercise where interacting differently with moral pain is practiced)]. More description of these ACT processes and how functional analysis can engage these processes is described in the section that follows.

In addition to in the moment intervention, functional analysis can also help the client identify how to intervene in future scenarios where moral pain is experienced. Through retrospective and in-the-moment chain analysis, future situations that are likely to trigger moral pain can be anticipated by the client and thus better support the deliberate practice of ACT processes and flexible responding. Once the pattern causing a person’s moral injury to persist is understood, specific parts of the chain that appear most consequential to maintaining moral injury can be targeted in treatment and matched with specific processes that might be more likely to disrupt the behavior maintaining moral injury. Detailed examples of how functional analysis can be used to facilitate ACT interventional processes are described at the end of each process section based on the chain analyses in Figures 2, 3.

#### Contact With the Present Moment

The present moment is the only place behavior change can occur. Therefore, clients must develop the ability to flexibly contact the present moment to become more aware and relate to their moral pain differently. Functional analysis can be used to help individuals open up to their experiences in the here and now by identifying behaviors (i.e., storytelling, humor) that move the client away from the present. Noticing client experiences in the here and now can also be helpful in understanding what is driving the target behavior. Slowing down and considering each link in the chain analysis can help reinforce the client’s experiences in the present moment, including noticing any urges to avoid or change those experiences. Content in the chain that reflects the client’s attention is focused on the past (e.g., memory of MIE) or future (e.g., worry about the reaction to future reminders of the MIE) can serve as a signal to observe experiences in the present moment (rather than ruminating about the past or future).

The Veteran case example (Figure 2) provides many opportunities to contact the present moment and disrupt the cycle maintaining moral injury (e.g., The Veteran could slow down and notice his experience related to any link in the chain). The triggering event might be one of the most efficient intervention points in the chain related to contacting the present moment. Knowing that his girlfriend’s child is a trigger for experiencing moral pain (and for his attention becoming consumed by past experiences), the Veteran could engage in a mindfulness practice focused on connecting with his experiences in the here and now and paying attention to what he experiences (including moral pain). With the case example of the health care provider (Figure 3), the triggering event may have been less obvious to the client as potentially provoking a pattern of behavior maintaining moral injury. Here, contact with the present moment might be facilitated in link 3 of the chain, working with the provider to notice her experience of contempt in the moment and any urges that come with experiencing contempt (e.g., urges to attack). Links 6 and 9 could also provide opportunities to slow down and notice her experiences in the here and now.

#### Defusion

When working with moral injury, thoughts, emotions, sensations, memories, and urges closely linked to an individual’s MIE can be particularly evocative. Learning to take the perspective of an observer of one’s moral pain is critical to interacting differently with that moral pain. Practicing observing thoughts, emotions, sensations, urges, and memories as an experience someone *has* rather than an experience they *are* can help individuals approach their moral pain. The more evocative the experience, the more applicable repeated practice of defusion is to learning to interact differently with that moral pain. Applying defusion to the content immediately precipitating the target behavior may be particularly impactful in helping the individual to choose alternatives to the target behavior that has facilitated avoidance or control of moral pain.

Early intervention through defusion could facilitate building new pathways of behavior that do not lead to the target behavior and instead are guided by the client’s values. For the Veteran case example (Figure 2) many of the links in the chain would be good opportunities to practice defusion. Defusion in chain link 3 from the thought “I’m a piece of shit” could be an important intervention point. An ACT exercise like “thoughts on cards” might help to facilitate perspective taking associated with the thought “I’m a piece of shit,” looking at this experience literally for what it is (a thought) rather than what the mind says it is (a truth) (44). Defusion from the thoughts and emotions indicated on links 4, 6, and 9 could also be practiced via thoughts on cards or other exercises that facilitate observing thoughts. Additionally, defusion could be applied to the urges evident in this chain. For instance, prior to lying to his girlfriend in link 7, the Veteran could have worked to defuse from this urge (e.g., watching the urge rise and fall in his body) and considered the extent to which it was consistent with who he wants to be in his relationship to lie to his girlfriend. Some of the most notable opportunities for defusion in the health care provider case example (Figure 3), to break the cycle of moral injury and potentially prevent the target behavior, include practicing observing the thought in link 5, “my boss is heartless and incompetent,” the emotion in links 3 and 6, and the urge to numb out in link 7. Different approaches to defusion could be taken with a guided experiential exercise, for example practicing labeling these thoughts, emotions, and urges as they arise (e.g., “I’m noticing I’m having the thought that ‘my boss is heartless and incompetent.”’ “I’m noticing I’m experiencing an urge to numb out and get away from this feeling”).

#### Acceptance

Part of avoiding, disengaging from, and attempting to control moral pain, typically involves being closed off to one’s internal experiences and to opportunities for reinforcement in the environment. Within ACT-MI, acceptance processes emphasize opening up to and making space for any internal experiences in the present moment, including moral pain, in the service of greater freedom to live values. Acceptance processes can be used across the chain to open up to one’s experiences and practice holding moral pain lightly. Facilitating acceptance of moral pain is particularly important from a functional contextual perspective because behavioral patterns linked to avoidance, control, and non-acceptance tend to maintain moral injury. Like defusion strategies, making room for the facets of moral pain that motivate the target behavior can be useful for shifting the individual’s relationship to their moral pain, and create opportunities for behavioral change.

In both case examples, contact with the present moment and defusion processes help to slow down and notice experiences in the present moment rather than automatically responding to these experiences through a pattern of behavior that maintains moral injury. Activating the acceptance process can facilitate not only noticing experiences, but a willingness to allow oneself to have these experiences. For the Veteran (Figure 2), acceptance of shame in links 6 and 9 could be helpful in cultivating a new pathway of behavior, informed by his values rather than avoidance of shame. This could include noticing how shame shows up in his body, physically opening up to that shame with a shift in body posture, and watching the emotion rise and fall. Practicing acceptance for the health care provider (Figure 3) might be particularly important at links 3, 6, and 7. Engaging in an exercise to practice relating to her moral pain and opening up to it, for instance “physicalizing” her emotions (imagining the shape, color, weight, temperature of her emotions while practicing willingness to gently hold the emotional object), could be helpful in breaking her pattern of “numbing” and disconnecting from her emotions (18).

#### Self as Context

There are typically multiple opportunities in the context of functional analysis to step back from particular narratives and practice perspective taking. Living inside of stories about what it means to have experienced a MIE can prevent living a life “outside” of those narratives, a life connected to meaning and purpose. If a behavioral chain is focused on an event that happened in the past, it can be useful to practice perspective taking related to temporality. For example, the client may be encouraged to notice that their conscious self extends across time. This conscious “observer” self was present before and during the MIE, during the target behavior, and now is present in session. This awareness of perspective across time allows the client to engage with the idea that while their experiences may shift moment to moment, their ability to hold these experiences, including moral pain, remains constant. The realization of a conscious self that is aware of, and therefore transcends, moral pain is also helpful in reinforcing to the client that although the MIE and moral pain is part of a person’s history or experience, it also need not define them as an individual.

Related to self as context, in the Veteran case example (Figure 2) link 4 in the chain, “I can’t be trusted around kids,” appears to be a story he generates to avoid the opportunity for exposure to moral pain that interacting with children will evoke. A self-compassion exercise could be helpful here, for the Veteran to practice holding this thought with kindness for the observer (himself) who experienced this thought and also the MIE in war. For the health care provider (Figure 3), engaging self as context related to her story about her emotions in link 7, “it’s best to turn off my emotions,” would be important for breaking the cycle of responding to her husband in anger. Exploring the historical contexts relevant to the development of this story (e.g., “Showing emotions is a sign of weakness” in the military or in health care settings) could be useful in practicing observations of experience, gaining distance from the most captivating facets of this narrative in the service of living values (rather than a life trapped in a story).

#### Values

A life guided by choice and what matters most to a person is impossible when their behavior is controlled by attempts to avoid moral pain. To break free from moral injury, it is critical to practice living one’s values especially in the presence of moral pain. Contacting such values, and the experiential sense of vitality that accompanies living them, can serve as a motivator for clients to interact differently with moral pain, to connect to something vital. Related to the long-term impacts of the target behavior, living a life attempting to avoid and control moral pain interferes with an individual’s ability to live their values. The triggering event can be a helpful starting point in identifying where an individual might consider living their values and even practice living them through committed action. Clients might be asked, “If you were to go back to this situation, what kinds of behaviors might align with your values?” The connection to values can also be made related to links in the chain containing a person’s moral pain. For instance, on the one hand, the experience of shame may signal that a client cares about behaving honorably relative to their community. On the other hand, anger may signal that an individual cares about fairness. Helping a person see that their moral pain is actually an indicator of their values can help facilitate motivation to notice and interact differently with that pain.

To break cycles of moral injury and live values, exploring the pain linked to values is critical. For the Veteran described in Figure 2 this would mean identifying the pain related to killing a child in war (link 3) and the pain associated with not being present for his girlfriend and her child (long-term impacts, box 12) and the values this pain indicates in both parts of the chain. This could be particularly important to actually living his values in these relationships. In the case example of the health care provider (Figure 3), values exploration could be facilitated through discussion of the long-term impacts of the target behavior. For instance, a provider might ask “How is it that you want to show up as a partner?, What matters most to you about your work?” It could also be helpful to slow down and reflect on values associated with the pain that shows up in links 3–6 and link 9.

#### Committed Action

Opportunities to live one’s values are present in every moment. Therefore, functional analysis can be used as an exercise to identify of opportunities for behavioral change, emphasizing directly changing behavior in response to moral pain in session. When collaboratively choosing committed actions with a client to disrupt a cycle of persistent moral injury, it is important to select small, clear, values-consistent behaviors that are seen as feasible to the client to engage even while experiencing moral pain. Over time, clients can progress to increasingly more significant changes in behavior. Engaging in behaviors consistent with one’s values in the face of moral pain often requires a degree of kindness for the experiencer (e.g., a willingness to do something kind for oneself even when experiencing thoughts like “I deserve to suffer”). Encouraging the client to practice self-compassion by gently holding in awareness the moral pain emerging in the chain analysis links may therefore be particularly useful in disrupting moral injury.

For both the Veteran and health care provider, small behaviors consistent with values can be engaged to literally practice building new patterns of behavior guided by values. For the Veteran, an example of this could be spending a short amount of time with his girlfriend and her son participating in an activity they all enjoy that involves minimal conversation (e.g., going to see a movie). This could be a first step in disrupting the pattern maintaining moral injury that is negatively impacting his relationship. He could engage in this behavior in the future, or in the context of the chain (e.g., he could plan an activity that he participates in with his girlfriend and her child in response to the thought “I can’t be trusted around kids”). In addition to overt behaviors that represent committed actions, covert behaviors can also facilitate committed action in the service of values. For example, if the Veteran engages in the physicalizing emotions exercise to interact differently with his shame, he is engaging flexibly with his emotions in the service of his values. Committed action for the health care provider (Figure 3) could take the form of having a conversation with her husband about her emotional experience associated with feeling triggered by his comment.

### Acceptance and Commitment Therapy for Moral Injury Summarized

Through strategies that activate the processes of contact with the present moment, defusion, acceptance, self as context, values, and committed action we hope to directly intervene on the behaviors that maintain moral injury by targeting their functions. If moral injury is maintained through negative reinforcement, we would work to use ACT-MI skills to help an individual relate differently to their moral pain rather than relying on the target behavior to provide escape. Related to positive reinforcement, we might encourage the client to practice noticing urges to change their experience (e.g., planning suicide to facilitate a sense of peace) and instead practice making space for moral pain. We might take the opportunity to intervene on moral injury at an environmental level using functional analysis as well and in so doing help alter the *consequences* maintaining moral injury. Working with an individual’s environment to respond differently to the target behavior (e.g., if the client asks his wife to watch his children because they evoke moral pain, instead working with the client and his family to keep the children in his environment) may directly alter the consequences of that behavior and disrupt the cycle of moral injury. In sum, if the target behavior can be identified and experiences related to moral pain observed and held gently to live a life driven by values, experiences in this network no longer require avoidance or control. Rather, the moral pain that had previously functioned as a cue to avoid and control, may instead be transformed into a cue to approach, connect, and more deeply live one’s values.

## Discussion

In the current article, we describe a functional contextual approach to assessing and intervening on moral injury as an alternative to a syndromal model of conceptualization. Functional analysis is a purely idiographic form of assessment, allowing the determination of the specific behaviors maintaining moral injury, the function these behaviors serve, and interventions to alter them. Because any behavior can serve the function of being used to avoid or control moral pain, an approach ensuring the behaviors most relevant to moral injury are identified may facilitate more efficient intervention. We provided the reader with a framework for applying functional analysis using retrospective chain analysis (Figures 2, 3) and in the moment abbreviated functional analysis. We discussed using ACT processes to intervene on moral injury to break patterns maintaining suffering. This approach to conceptualizing moral injury is meant to target the mechanisms maintaining suffering and to empower individuals to build new patterns of behavior in response to moral pain that are guided by their values (rather than being limited to avoidance and control).

### Limitations

While there are significant benefits to a functional contextual approach to conceptualizing moral injury, there are also limitations. First, our approach to treating moral injury is incongruent with the assumption that pain must be reduced to live a meaningful life. Working with an individual to challenge and reduce their experience of shame could be helpful for some clients. However, this stance, even if presented unintentionally, could be invalidating to others who might firmly believe that shame should be felt for violating their morals. Challenging this experience, or even working to decrease this emotion, could communicate that we as clinicians are moral authorities telling a client that their experience is wrong. Many clients have found our approach to working with moral pain validating because the individual’s rationale for their moral pain is not challenged. Instead of treating moral pain as a symptom of moral injury that needs to be reduced, the ACT-MI clinician targets one’s behavior in response to their moral pain, as this behavior is maintaining the moral injury and related impairment in psychosocial functioning. The goal is to live well instead of feel good and in living well people tend to feel better [Gloster et al. (45)]. Learning skills to live with psychological pain in the service of one’s values, has been shown to indirectly result in decreases in moral distress, suggesting that skills cultivating the willingness to have moral pain may indirectly result in a reduction of that moral pain [Gloster et al. (45)]. However, it could be the case that this theoretical approach does not fit for a particular client who is solely focused on wanting to reduce their moral pain. If the client remains committed to pain reduction after addressing the problem of internal control and its costs and is still seeking to feel good rather than live well, another treatment approach for moral injury might be indicated.

Second, a goal of a syndromal approach to classification concerns a standard demarcation of disorder form non-disorder. If moral injury becomes a diagnostic category, relying on a purely functional contextual model would pose difficulties for billing and insurance purposes. Relying on functional analysis does not directly allow comparing an individual’s treatment progress to another individual with similar characteristics.

Third, it has also been historically challenging to research idiographic assessment measures, although this has become more accessible with methods like network analysis (12, 46, 47) and ecological momentary assessment procedures (12, 48). Investigators in the contextual behavioral science community have been working to develop measures and methods that can be more readily researched to facilitate functional analysis (46, 47).

### Future Directions

While our approach to case conceptualizing is based on research supporting behavior analysis and ACT, more research is needed to understand how this approach facilitates breaking patterns of moral injury and enhancing client engagement with meaning and purpose. We are currently conducting a randomized controlled acceptability and feasibility trial of ACT-MI, and in the context of this study, we are also testing the acceptability of our approach to case conceptualizing across treatment, with feedback from participants thus far being positive (i.e., from completed qualitative interviews). Future directions for research include investigating the extent to which this approach to case conceptualizing is efficacious. This could include understanding if sessions devoted to case conceptualizing across ACT-MI bolster the efficacy of the intervention in disrupting moral injury through outcomes related to functioning and values-based living. Additionally new formal measures are being developed to facilitate process-based assessment. Future studies should include an investigation of the Process Based Assessment Tool’s (PBAT) relevance in identifying the processes maintaining suffering associated with moral injury (47). The PBAT is a longitudinal assessment tool that includes process targets that are theoretically relevant to dimensions that maintain moral injury (affect, cognition, attention, social connection, motivation, overt behavior, and physical behavior) (47). Additionally, other repeated measures approaches using methodologies like ecological momentary assessment might help capture the processes maintaining moral injury at the level of individual clients. The incorporation of these assessment strategies may not only help to establish the utility of a functional contextual approach to moral injury, but may also expand the field’s understanding of process-based interventions for human suffering.

## Author Contributions

All authors contributed to the conceptualization, writing, and editing of the manuscript.

## Author Disclaimer

The views expressed are those of the authors and do not necessarily represent the views or policy of the VA or the United States Government.

## Conflict of Interest

The authors declare that the research was conducted in the absence of any commercial or financial relationships that could be construed as a potential conflict of interest.

## Publisher’s Note

All claims expressed in this article are solely those of the authors and do not necessarily represent those of their affiliated organizations, or those of the publisher, the editors and the reviewers. Any product that may be evaluated in this article, or claim that may be made by its manufacturer, is not guaranteed or endorsed by the publisher.
